# Is Response to Genotoxic Stress Similar in Populations of African and European Ancestry? A Study of Dose-Response After *in vitro* Irradiation

**DOI:** 10.3389/fgene.2021.657999

**Published:** 2021-11-11

**Authors:** Mamadou Soumboundou, Julien Dossou, Yossef Kalaga, Innocent Nkengurutse, Ibrahima Faye, Albert Guingani, Macoura Gadji, Koudbi J. Yameogo, Henri Zongo, Gora Mbaye, Ahmadou Dem, Mounibé Diarra, Rached Adjibade, Catherine Djebou, Steffen Junker, Noufissa Oudrhiri, William M. Hempel, Alain Dieterlen, Eric Jeandidier, Patrice Carde, Elie El Maalouf, Bruno Colicchio, Annelise Bennaceur-Griscelli, Michael Fenech, Philippe Voisin, Claire Rodriguez-Lafrasse, Radhia M’Kacher

**Affiliations:** ^1^Laboratoire Biophysique UFR-Santé, Dakar, Senegal; ^2^Département du Génie d’Imagerie Médicale et Radiobiologie, Cotonou, Benin; ^3^Centre Hospitalier Yalgado Radioprotection-Radiobiologie, Ouagadougou, Burkina Faso; ^4^Laboratoire de Radiobiologie, Bujumbura, Burundi; ^5^Cell Environment, DNA Damage R&D, Paris, France; ^6^Service Hématologie UCAD, Dakar, Senegal; ^7^Institute of Biomedicine, University of Aarhus, Aarhus, Denmark; ^8^APHP-Service d’Hématologie – Oncohématologie Moléculaire et Cytogénétique Hôpital Paul Brousse Université Paris Saclay/Inserm UMR 935, Villejuif, France; ^9^IRIMAS, Institut de Recherche en Informatique, Mathématiques, Automatique et Signal, Université de Haute-Alsace, Mulhouse, France; ^10^Service de Génétique Groupe Hospitalier de la Région de Mulhouse et Sud Alsace, Mulhouse, France; ^11^Department of Hematology, Gustave Roussy Cancer Campus, Villejuif, France; ^12^School of Pharmacy and Medical Sciences, University of South Australia, Adelaide, SA, Australia; ^13^Genome Health Foundation, North Brighton, SA, Australia; ^14^Universiti Kebangsaan Malaysia, Kuala Lumpur, Malaysia; ^15^Laboratoire de Radiobiologie Cellulaire et Moléculaire, Faculté de Médecine Lyon-Sud, UMR CNRS5822/IN2P3, IPNL, PRISME, Oullins, France

**Keywords:** African donors, European donors, irradiation, dicentric chromosome, telomeres, centromeres

## Abstract

**Background:** Exposure to genotoxic stress such as radiation is an important public health issue affecting a large population. The necessity of analyzing cytogenetic effects of such exposure is related to the need to estimate the associated risk. Cytogenetic biological dosimetry is based on the relationship between the absorbed dose and the frequency of scored chromosomal aberrations. The influence of confounding factors on radiation response is a topical issue. The role of ethnicity is unclear. Here, we compared the dose-response curves obtained after irradiation of circulating lymphocytes from healthy donors of African and European ancestry.

**Materials and Methods:** Blood samples from six Africans living in Africa, five Africans living in Europe, and five Caucasians living in Europe were exposed to various doses (0–4 Gy) of X-rays at a dose-rate of 0.1 Gy/min using an X-RAD320 irradiator. A validated cohort composed of 14 healthy Africans living in three African countries was included and blood samples were irradiated using the same protocols. Blood lymphocytes were cultured for 48 h and chromosomal aberrations scored during the first mitosis by telomere and centromere staining. The distribution of dicentric chromosomes was determined and the Kruskal-Wallis test was used to compare the dose-response curves of the two populations.

**Results:** No spontaneous dicentric chromosomes were detected in African donors, thus establishing a very low background of unstable chromosomal aberrations relative to the European population. There was a significant difference in the dose response curves between native African and European donors. At 4 Gy, African donors showed a significantly lower frequency of dicentric chromosomes (*p* = 8.65 10^–17^), centric rings (*p* = 4.0310^–14^), and resulting double-strand-breaks (DSB) (*p* = 1.32 10^–18^) than European donors. In addition, a significant difference was found between African donors living in Europe and Africans living in Africa.

**Conclusion:** This is the first study to demonstrate the important role of ethnic and environmental factors that may epigenetically influence the response to irradiation. It will be necessary to establish country-of-origen-specific dose response curves to practice precise and adequate biological dosimetry. This work opens new perspective for the comparison of treatments based on genotoxic agents, such as irradiation.

## Introduction

Exposure to genotoxic stress, such as ionizing radiation, chemical agents or viral infections is an inevitable by-product of modern life (McLean and Adlen, 2017; Masjedi et al., 2020). X-rays are widely used in industry, as well as in healthcare (Power et al., 2016). Vast numbers of epidemiological studies have reported the effects of exposure to naturally occurring, accidental, and medical radiation, especially on the incidence of late complications (Pernot et al., 2012). Biological signatures of ionizing radiation have been widely investigated (IAEA, 2011; Voisin, 2015). Thus, cytogenetic biological dosimetry is now considered an important tool for estimating the dose, not only after nuclear accidents (Edwards et al., 2004; Harada et al., 2014), but also in the treatment of cancer patients, for whom physical dosimetry is difficult to apply (M’Kacher et al., 1996, 1997, 1998).

Scoring of dicentric chromosome is considered a gold standard technique in cytogenetic biological dosimetry, is medically and legally recognized, and it still the most precise and sensitive method (IAEA, 2011). The recent introduction of telomere and centromere staining for scoring unstable chromosome aberrations has led to improved identification of aberrations and, moreover, rendered the analysis operator-independent through automated scoring technology (M’Kacher et al., 2014, 2015).

The major role of genetic factors in the response to genotoxic agents, such as ionizing radiation or viral infection, has been previously studied (M’Kacher et al., 2010; Kaser, 2020). Specific differences in the prevalence of certain cancers and pathologies have been observed between human populations (Blackman et al., 2018; Kaser, 2020; Liu et al., 2020; Lopes et al., 2020). Ethnicity is a complex entity composed of genetic background and environmental factors, such as diet, smoking, pollution, and infections. The involvement of ethnicity in the response to genotoxic stress, such as viral infection, has been described recently (Geoffroy et al., 2020). Very few studies have been devoted to ethnicity in relation to the response to ionizing radiation (Sapkota et al., 2020). Moreover, standardization of the norms of radiation therapy and protection does not take into account the ethnicity or genetic, environmental, or epigenetic factors that may cause inter-individual differences in response to radiation at the cytogenetic level. Many biological dosimetry laboratories have used the dose-response curve established by the International Atomic Energy Agency (IAEA) without taking into account the specificity of each population or the technical conditions of the laboratory (IAEA, 2011; Oestreicher et al., 2017). Of note, the RENEB network demonstrated significant differences in the established dose response curves in various participating laboratories. However, such differences are less striking when the estimated doses are considered, allowing the examination of intrinsic and extrinsic factors in the estimation of dose (Oestreicher et al., 2017).

Here, as a working hypothesis we studied the effect of ethnicity on the *in vitro* response to ionizing radiation. We analyzed the induction of unstable chromosomal aberrations in circulating lymphocytes in three populations: six healthy donors of African ancestry living in Africa (Senegal), five healthy donors of African ancestry (Senegal) living in Europe (France), and five healthy Caucasian donors living in Europe. Dose-response curves were established for each group. Significant differences were found between the dose response-curves of healthy African and European donors. To validate these data, three other cohorts of healthy donors living in three different African countries were included. The donors of African ancestry living in Europe demonstrated a response that was intermediate relative to the other groups. These *in vitro* data underscore the necessity to establish a specific dose-response curve for each biological dosimetry laboratory. Overall, these data raise the possible role of ethnicity, as well as environmental factors, in the response to genotoxic stress.

## Materials and Methods

### Blood Samples

Peripheral blood samples were obtained from the individuals in each of the three sub-groups. The first group consisted of six healthy donors of African ancestry living in Senegal [3 women/3 men, mean age 31 years (19–34)]. The second group of healthy donors from African ancestry, living in Europe, consisted of five donors [3 men, 2 women, mean age 37 years (29–49 years)]. The third group consisted of five healthy Caucasian donors living in Europe [1 man, 4 women, mean age 44 years (31–61 years)]. The validation group was composed of six healthy donors from Benin [2 women/4 men, mean age 25 years (20–35)], four from Burundi [1 women/3 male, mean age 31 years (26–35)], five from Burkina Faso [2 women/3 men, mean age 32 years (30–35)].

None of the donors had ever received radiation therapy and they were free of viral infections (HIV, Hepatitis B and C, THA etc.). The African samples were shipped to France via DHL in less than three days. The same protocol was applied to European samples (irradiation and culture after 3 days).

All samples were irradiated using an X-RAD320 (Lyon-Sud). Lymphocyte culture, slide preparation, telomere and centromere staining, as well as analysis of the results, were performed in the Cell Environment Laboratory (Paris) under identical conditions.

All donors provided informed consent for the collection of blood samples and the project was approved by the ethics committees of each country (Protocol 057/2015/Cer/UCAD from Senegal; Protocol 012/2017 from Burundi; and Protocol 006/2017 from Benin).

### Irradiation Procedure and Metaphase Preparation

Blood lymphocytes were exposed to various doses of X-rays (0, 0.5, 1, 2, and 4 Gy) using an X-RAD320 (Lyon-Sud) at a dose-rate of 0.1 Gy/min, with a maximum photon energy of 250 kV (250 kV accelerating potential, X-ray tube type comet; PXi (PRECISION X-RAY irradiation), North Branford, US). Subsequently, the blood samples were cultured for 48 h in RPMI 1640 (Gibco, Grand Islands, NY) supplemented with 10% fetal calf serum (Eurobio, Courtaboeuf, France) and 1% antibiotics (Gibco, Grand Island, NY, United States). T lymphocytes were stimulated with phytohemagglutinin form M (Gibco, Grand Island, NY, United States). BrdU was added to differentiate between the first and second mitotic divisions. Colcemid (Gibco KaryoMAX, France) was added 2 h before the cells were harvested. Metaphases were prepared using standard procedures, and the slides were stored at −20°C until use.

### Telomere and Centromere Staining

Telomeres and centromeres were stained using the Q-FISH technique with a Cy-3-labeled PNA probe specific for TTAGGG for telomeres and a FITC-labeled PNA probe specific for centromere sequences (both from Eurogenetec, Liege, Belgium), as previously described (M’Kacher et al., 2014). Briefly, slides were washed with 1X PBS for 5 min at room temperature and fixed in 4% formaldehyde for 2 min at room temperature. Following three rinses with PBS for 5 min each, the slides were treated for 7 min with pepsin (0.5 mg/ml; Sigma, France) at 37°C. The slides were washed again in PBS and then re-fixed in 4% formaldehyde for 2 min. After three PBS washes of 5 min each, the slides were sequentially dehydrated with 50, 70, and 100% ethanol and air-dried. A solution (50 μl) containing the PNA probes (telomere and centromere, 0.3 μg/ml each) was added to the slides, and they were subsequently denatured on a hot plate at 80°C for 3 min and incubated in the dark for 1 h at room temperature. After hybridization, slides were washed (3 × 5 min) with 70% formamide/10 mM Tris pH 7.2 and then in 50 mM Tris pH 7.2/150 mM NaCl pH 7.5/0.05% Tween-20 (3 × 5 min). After washing with PBS, the slides were counterstained with DAPI and mounted with PPD. Images of metaphase cells were acquired using automated acquisition module Autocapt software (MetaSystems, version 3.9.1) using a ZEISS Plan-Apochromat 63 × /1.40 oil and CoolCube 1 Digital High Resolution CCD Camera. The analysis was performed using ChromScore software (Cell Environment).

### Scoring of Chromosomal Aberrations

**FIGURE 1:**
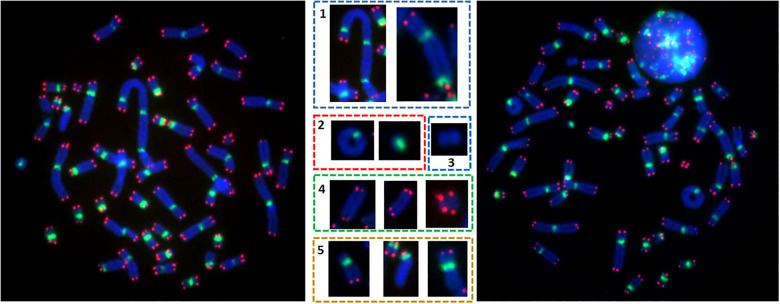
Metaphases after telomere (red) and centromere (green) staining permit precise detection of unstable chromosomal aberrations e.g., the presence of (1) dicentric chromosomes, (2) ring chromosomes, (3) acentric rings, (4) acentric chromosomes with 4 red signals, and (5) telomere deletion.

Two slides per dose of irradiation were used to analyze unstable chromosomal aberrations after telomere and centromere staining. For each dose, an average of 100 metaphases was counted on each slide. Only metaphases with 46 centromeres were analyzed. Chromosomal aberrations were scored according to the presence or absence of telomere and centromere sequences (Kaddour et al., 2017). We detected dicentrics, centric and acentric rings, as well as fragments with four telomeres (ace(+/+)), resulting from a fusion event generally accompanied by the formation of a dicentric chromosome or centric ring. We also detected fragments with two telomeres (ace(±)), representing terminal deletions, as well as acentric fragments without telomeres (ace(−/−)), representing interstitial deletions (Figure 1). Telomere deletions (i.e., loss of two telomeres in the same arm) were also detected. The combined information scored by telomere and centromere staining allowed us to precisely calculate the number of unrepaired or mis-repaired double strand breaks (DSB) that generated the chromosomal aberrations: dicentric chromosomes and centric rings with a fragment containing four telomeres were considered as two DSB. Excess acentric fragments were considered to be the result of one DSB for terminal deletions, with only two telomeres and two DSB for interstitial deletion fragments with no telomere sequences. Telomere deletion was considered the result from one DSB.

### Statistical Analysis

A script in R^®^ was developed according to the IAEA recommendations (IAEA, 2011). The curves generated in this study are based on generalized linear models using the glm function of the standard Stats package, computing the maximum likelihood estimation, given the count of aberrations following a quasi-Poisson distribution. The curves were fitted as a quadratic function alpha^∗^x + beta^∗^x^2^ + C. The standard error of the mean (SEM) is shown and the dose-response curve is plotted in gray. The uncertaintly of the aberration count is plotted as error bars according to the exact Poisson 95% confidence intervals (Johnson et al., 2005). Uncertainty bounds of the dose given by the aberration count can be estimated using Merkle’s method, combining the variance-covariance matrix of the fitted function and the confidence intervals of the aberration counts. Thus, the variance-covariance values are also presented.

## Results

### Spontaneous Unstable Chromosomal Aberrations

Following telomere and centromere staining, the frequency of unstable chromosomal aberrations was analyzed in healthy African and European donors. Prior to irradiation, no dicentric chromosomes were found in any of the healthy African donors (living in Africa or in Europe) after scoring 12,450 metaphases in the first mitotic division during culture. Only a few acentric chromosomes and telomere deletions were identified (0.17% DSB). The incidence of dicentric chromosomes in Caucasian donors was six dicentric chromosomes after scoring 5,200 metaphases. Thus, a significant difference was observed between the frequency of dicentric chromosomes (*p* < 10^–9^) and resulting DSB (*p* < 10^–16^) in circulating lymphocytes of healthy African and Caucasian donors.

### Significant Difference in the Dose Response Curves

After X-ray exposure of circulating lymphocytes *in vitro* and scoring of chromosomal aberrations in the first mitotic metaphases using telomere and centromere staining, linear quadratic dose response curves were obtained using dicentric chromosomes, dicentric and ring chromosomes, and calculated DSB, taking into account all scored aberrations. The distribution of dicentric chromosomes per cell and their associated σ2/Y and U value obtained from 0 to 4 Gy were calculated. We observed a Poisson distribution at each dose. Over dispersion was only observed for 4 Gy. The curve-fit coefficients for all dose-response curves are shown in Table 1.

**TABLE 1:** α and β coefficients for different groups after X-ray exposure and scoring of dicentric chromosomes, dicentric and ring chromosomes, and the calculated DSBs following telomere and centromere staining.

Scored aberrations	Group	α ± SE	β ± SE	α/β	cov(α,β)
Dicentrics	African ancestry (Senegal) living in Africa	0.168 ± 0.018	0.061 ± 0.006	2.75	–0.0001
	All African ancestry living in Africa	0.103 ± 0.008	0.079 ± 0.002	1.30	−2.210^−5^
	African ancestry (Senegal) living in Europe	0.084 ± 0.022	0.122 ± 0.007	0.68	–0.0001
	Caucasian living in Europe	0.147 ± 0.04	0.128 ± 0.013	1.14	–0.0005
Dicentrics rings	African ancestry (Senegal) living in Africa	0.207 ± 0.065	0.065 ± 0.008	3.18	–0.0002
	All African ancestry living in Africa	0.107 ± 0.010	0.102 ± 0.003	1.04	−3.10^−5^
	African ancestry (Senegal) living in Europe	0.083 ± 0.034	0.146 ± 0.011	0.56	–0.0003
	Caucasian living in Europe	0.120 ± 0.043	0.171 ± 0.013	0.70	–0.0005
DSBs	African ancestry (Senegal) living in Africa	0.512 ± 0.050	0.155 ± 0.016	3.30	–0.0007
	All African ancestry living in Africa	0.263 ± 0.026	0.257 ± 0.009	1.02	–0.0024
	African ancestry (Senegal) living in Europe	0.219 ± 0.111	0.357 ± 0.369	0.61	–0.0038
	Caucasian living in Europe	0.277 ± 0.091	0.257 ± 0.009	1.07	–0.0002

*Standard errors and covariance α/β were shown.*

**FIGURE 2:**
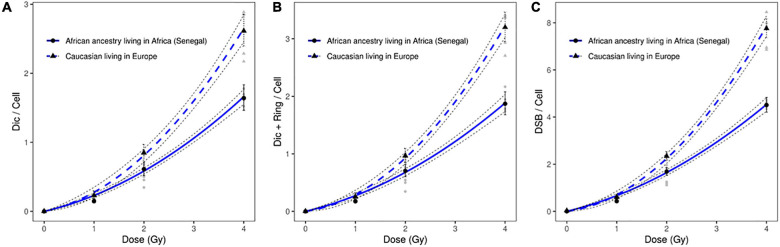
Dose response curves obtained after X-ray irradiation of circulating lymphocytes *in vitro* of healthy donors of African ancestry living in Africa (Senegal) and European ancestry living in Europe after scoring of **(A)** dicentric chromosomes **(B)** dicentric and ring chromosomes, and **(C)** calculated DSBs. The mean numbers of chromosomal aberrations with 95% confidence intervals of each population are shown. Individual values for each of the donors are also plotted, as well as the standard errors (dotted line).

Significant differences were observed between the dose response curves obtained after the scoring of induced dicentric chromosomes in peripheral blood lymphocytes of six healthy African donors (Senegal) relative to healthy Caucasian donors (Figure 2A). Similar results were obtained after the scoring of dicentric and ring chromosomes (Figure 2B). The observed difference between the frequencies of estimated DSB following the exposure of circulating lymphocytes of healthy African donors (Senegal) relative to healthy Caucasian donors (France) was even greater than that based on scoring of dicentric chromosomes alone (Figure 2C).

**FIGURE 3:**
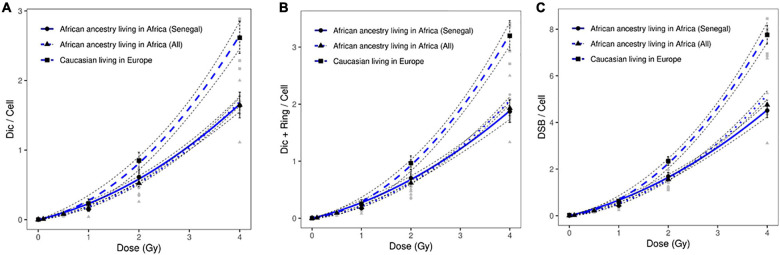
Dose response curves obtained after X-ray irradiation of circulating lymphocytes in vitro of healthy donors of all African ancestry living in Africa (Senegal, Benin, Burkina Faso and Burindi) and European ancestry living in Europe after scoring of **(A)** dicentric chromosomes, **(B)** dicentric and ring chromosomes, and **(C)** calculated DSBs. The mean numbers of chromosomal aberrations with 95% confidence intervals of each population are shown. Individual values for each of the donors are also plotted, as well as the standard errors (dotted line). No significant difference was observed between the dose response curve obtained healthy Senegalese donors and all healthy African donors.

To validate these data, we introduced healthy African donors from three different countries: Benin, Burkina Baso, and Burindi. The dose response curves obtained from healthy Caucasian and all healthy African donors are presented in Figure 3. Significant differences were observed after the scoring of dicentric chromosomes (Figure 3A), dicentric and ring chromosomes (Figure 3B), and the resulting DSBs (Figure 3C).

After X-ray exposure at 4 Gy, circulating lymphocytes of healthy African donors showed a significantly lower frequency of dicentric chromosomes (*p* = 8.65 10^–17^), centric rings (*p* = 4.0310^–14^), and total DSBs (*p* = 1.32 10^–18^) than those of healthy Caucasian donors (Figures 2, 3).

### Environmental Factors in Response to *in vitro* Irradiation

**FIGURE 4:**
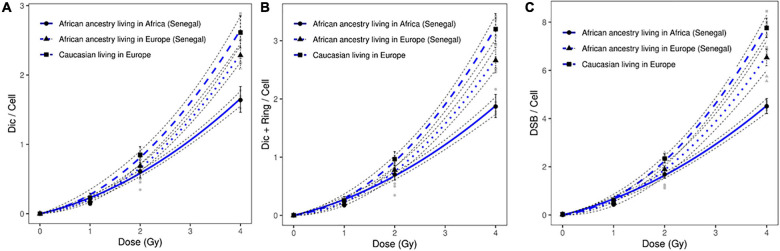
Dose response curves obtained after X-ray irradiation of circulating lymphocytes in vitro of healthy donors of African ancestry living in Africa (Senegal), healthy donors of African ancestry (Senegal) living in Europe, and European ancestry living in Europe after scoring of **(A)** dicentric chromosomes, **(B)** dicentric and ring chromosomes, and **(C)** calculated DSBs. The mean numbers of chromosomal aberrations with 95% confidence intervals of each population are shown. Individual values for each of the donors are also plotted, as well as the standard errors (dotted line).

In this study, we irradiated circulating lymphocytes of healthy donors of African ancestry living in Europe (France). We observed a significant difference in the frequency of induced chromosomal aberrations between healthy donors of African ancestry (Senegal) living in Europe (France) and healthy donors of African ancestry living in Africa (Senegal). This difference was observed after analysis of all induced chromosomal aberrations: dicentric chromosomes (*p* = 1.72 10^–4^); ring chromosomes (*p* = 3.810^–3^); and DSBs (*p* = 6.36 10^–9^) after 4 Gy of *in vitro* irradiation. These data suggest the contribution of environmental factors in the response to genotoxic agents such as ionizing irradiation (Figure 4).

In addition, a significant difference in the frequency of induced chromosomal aberrations was observed between healthy donors of African ancestry living in Europe and Caucasian donors living in Europe (dicentric chromosomes: *p* = 1.9710^–2^; ring chromosomes: 4.3510^–4^; and DSBs: *p* = 2.89 10^–2^) (Figure 4).

## Discussion

The contribution of confounding factors in the response to genotoxic stress of populations is an emerging topical issue (Kaser, 2020). The heterogeneity observed in the response to exposure to genotoxic agents raises the question of genetic susceptibility in the response in terms of morbidity and mortality in different populations. Ethnic disparities have been associated with variable survival outcomes in solid malignancies, including pancreatic, neuroendocrine, prostate, colorectal, and female breast neoplasms (Goksu et al., 2020). To date, only a few studies have investigated the impact of ethnicity on the radiation response *in vitro* and *in vivo* (Sigurdson et al., 2008). The primary objective of this study was to determine a putative relationship between ethnicity and the response to exposure to genotoxic stress such as irradiation.

We identified significant ethnic differences concerning the spontaneous frequency of unstable chromosomal aberrations. Healthy Caucasian donors living in Europe exhibited a higher rate of dicentric chromosomes than healthy African donors, irrespective of the country they lived in. In addition, the global rate of unstable chromosomal aberrations observed in the Caucasian cohort was significantly higher than the rate previously published (IAEA, 2011; Voisin, 2015). The use of more sensitive techniques for the detection of chromosomal aberrations, such as telomere and centromere staining by itself, cannot explain such a difference. Indeed, we speculate that the background of these aberrations in the general population in Europe is most probably higher due to the various sources of ionizing irradiation exposure related to modern life in this country (France). A re-evaluation of the background of these aberrations in large cohorts of healthy populations using a sensitive technique may establish the relationship between environmental exposure and the increasing frequency of these aberrations. Of note, there was a difference in the mean age of donors living in Africa and the healthy Caucasian donors. However, previous studies have demonstrated that the frequency of dicentric chromosomes is more stable in this age range (20–69 years) (Sigurdson et al., 2008). Future studies on a large cohort of healthy African and Caucasian donors should address this difference using cohorts of similar age.

First, we compared the frequency of unstable chromosomal aberrations after *in vitro* irradiation of circulating lymphocytes of six donors of African ancestry living in Africa (Senegal) and five Caucasian donors living in Europe (France). Significant differences were observed between the frequencies of dicentric chromosomes, centric ring chromosomes, and calculated DSBs in the circulating lymphocytes of healthy donors of African ancestry relative to those in the Caucasian cohort. The addition of three other cohorts of healthy donors of ancestry living in Africa allowed the validation of our data. These findings shed light on the important contribution of ethnicity in the response to genotoxic agents such as irradiation. Dicentric chromosomes are considered to be the best and most sensitive biomarker of irradiation. The frequency of induced dicentric chromosomes in healthy adult donors (20–69 years) has been proposed to be independent of many confounding factors, such as age (Bakhmutsky et al., 2014). This is why the detection of dicentric chromosomes is considered to be the gold standard (IAEA, 2011; Voisin, 2015). However, there are currently no published data on the effect of aging on radiation-induced dicentrics in the age range of the groups investigated in our study (i.e., 19–61 years). The mean age of the Caucasian cohort (44 years) and African cohorts (31, 37, 25, 31, 32 years) differs by a margin of 7–19 years, which may or may not be large enough to influence the data, assuming the existence of an age effect. The only data available for radiation-induced dicentrics is a study comparing neonates, children, and adults (mean age, 0, 2.5, 39.5 years), which showed a significant increase in radiation-induced dicentrics in adults relative to children and neonates *in vitro* using a challenge dose of 978 milliGray CT radiation (Gomolka et al., 2018). Therefore, current evidence indicates an age-difference of at least 37 years is required to observe the age-effect, and, therefore, the probability that age was a major confounding factor in our study is relatively small, because the age difference between our groups was only 7–19 years.

In addition, the frequency of induced ring chromosomes appeared to more highly effected by ethnicity. The F-ratio (the ratio of dicentric chromosomes to centric ring chromosomes) observed in healthy Caucasians living in Europe was approximately 5%. This ratio is in concordance with those described previously (Kaddour et al., 2017). In healthy donors of African ancestry, the frequency of ring chromosomes was significantly lower than that in the Caucasian cohort. Similarly, the frequencies of acentric chromosomes related to terminal deletion, as well as chromosomal deletions, were significantly different between the two populations. These data suggest that the observed differences are not only quantitative but also qualitative. It will be interesting to analyze DNA repair mechanisms in these two populations.

Next, we analyzed the putative critical role of environmental factors in the response to genotoxic agents. We scored induced aberrations after the irradiation of circulating lymphocytes of healthy donors of African ancestry living in Africa and those living in Europe (France). Significant differences were observed between these two cohorts, suggesting a role of environmental factors in the response to irradiation and modification of DNA repair mechanisms.

Differences in chromosomal aberrations between donors living in different countries may also be explained by differences in food intake, which affects the nutritional status. For example, folate and vitamin B-12 deficiencies have been shown to increase chromosome fragility *in vivo* (Fenech, 2012) and inadequate folate or zinc concentrations, at physiological levels, increase sensitivity to the DNA damaging effects of ionizing radiation *in vitro* as measured using chromosomal instability biomarkers, such as micronuclei (which originate from acentric chromosome fragments or whole chromosomes with defective centromeres) and nucleoplasmic bridges (which originate from dicentric chromosomes) (Beetstra et al., 2005). In this regard, it is pertinent to note that folate and vitamin B-12 deficiency also exert epigenetic effects by limiting the synthesis of the methionine and S-adenosyl methionine required for cytosine methylation, which is essential for the epigenetic control of gene expression and structural integrity of centromeres (Fenech, 2012). In addition, analysis of telomere length and instability could advance our knowledge of the epigenetic origins of the differences observed in our three cohorts (M’Kacher et al., 2007).

In this study, we demonstrate, for the first time, a significant difference in the frequency of induced unstable chromosomal aberrations in populations of different ethnic origin. The cohorts used in this study did not allow us to confirm the role of ethnicity in the response to irradiation. Although preliminary, our results encourage further studies to validate these findings in a larger cohort of healthy donors of different ethnicity, gender, nutritional status, and age. Our data suggest a possible crucial impact of ethnicity, not only in biological dosimetry and estimation of the dose after exposure to genotoxic agents, but also in radiation therapy of cancer patients.

## Conclusion

Here, we demonstrate a significant difference in the dose response to irradiation of circulating lymphocytes *in vitro* from healthy donors of African ancestry, irrespective of their country of residence, and Caucasians living in Europe (France). Interestingly, the dose response curves for the healthy donors of African ancestry living in Europe also showed significant differences from those of Africans living in Africa, as well as those of healthy donors of European ancestry, suggesting that environmental factors may have a substantial influence on the response to the genotoxic effects of radiation. These differences, which were identified by scoring unstable chromosomal aberrations using telomere and centromere staining, suggest that genetic and epigenetic factors contribute to radiation responses. Supplementary investigations with a large cohort are required to validate our cytogenetic findings and shed light on the molecular mechanisms of genotoxic agents that affect the health of genetically and epigenetically different populations, e.g., of different ethnicity and country of residence.

## Data Availability Statement

The original contributions presented in the study are included in the article/supplementary material, further inquiries can be directed to the corresponding author/s.

## Ethics Statement

The studies involving human participants were reviewed and approved by Protocol 057/2015/Cer/UCAD from Senegal; Protocol 012/2017 from Burundi; and Protocol 006/2017 from Benin. The patients/participants provided their written informed consent to participate in this study.

## Author Contributions

RM’K, MS, and CR-L: conceptualization. RM’K, MS, JD, YK, IN, AG, HZ, IF, JY, and BC: methodology. GM, MD, RA, CD, NO, ADe, EJ, EE, and PC: formal analysis and investigation. RM’K, PC, and PV: writing–original draft preparation. PV, MF, PC, WH, SJ, EJ, AB-G, and RM’K: writing–review and editing. All authors have read and agreed to the published version of the manuscript.

## Conflict of Interest

The authors declare that the research was conducted in the absence of any commercial or financial relationships that could be construed as a potential conflict of interest.

## Publisher’s Note

All claims expressed in this article are solely those of the authors and do not necessarily represent those of their affiliated organizations, or those of the publisher, the editors and the reviewers. Any product that may be evaluated in this article, or claim that may be made by its manufacturer, is not guaranteed or endorsed by the publisher.
